# Spontaneous Internal Fistulization of a Pancreatic Pseudocyst Into the Duodenum: A Case Report

**DOI:** 10.7759/cureus.88522

**Published:** 2025-07-22

**Authors:** Hanane Aksim, Oualid Haddadia, Meryem Belhamdiya, Rachid Akka

**Affiliations:** 1 Gastroenterology and Hepatology, Military Hospital of Avicenne, Marrakesh, MAR; 2 Hepatogastroenterology, Military Hospital of Avicenne, Marrakesh, MAR

**Keywords:** duodenum, endoscopy, fistula, pancreas, pseudocyst

## Abstract

Pancreatic pseudocysts are organized fluid collections without epithelial lining, usually containing pancreatic juice or liquefied necrotic tissue. Advances in imaging techniques, particularly ultrasound and computed tomography, have significantly improved the diagnosis and management of these cystic lesions. In recent years, minimally invasive approaches such as endoscopic drainage and interventional radiology have become important therapeutic alternatives. Laparoscopic cystodigestive anastomosis is also increasingly used in selected cases. We report a rare case of spontaneous fistulization of a pancreatic pseudocyst into the duodenum. The diagnosis of such fistulization remains challenging due to its rarity and the absence of specific symptoms. This case highlights the possibility of managing spontaneous pancreatic pseudocyst fistulization using endoscopic techniques alone, without the need for surgical intervention. This minimally invasive approach emphasizes the importance of endoscopy in the management of complex pancreatic fluid collections.

## Introduction

Cystic lesions of the pancreas, including various types, may be present in up to 49.5% of the general population [1]. Pancreatic pseudocysts, a subset of these lesions, can rarely lead to spontaneous fistulization. Perforations or fistulization of pseudocysts into organs such as the stomach, colon, duodenum, peritoneal cavity, pleural cavity, portal vein, and even through the abdominal wall have been reported in less than 3% of cases [1]. In cases of strong clinical suspicion of pseudocyst fistulization, early computed tomography (CT) imaging is essential for rapid diagnosis [2]. However, when CT is inconclusive or unavailable, endoscopy plays a pivotal role in localizing the site of fistulization [3]. We report the case of a pancreatic pseudocyst revealed during an etiological workup of acute atypical epigastralgia associated with bilious vomiting, which fistulated spontaneously into the duodenum and was successfully treated endoscopically.

## Case presentation

We report the case of a 58-year-old adult male with no specific pathological history, including no prior episodes of pancreatitis, alcohol use, or trauma. He presented with atypical acute epigastric pain of moderate intensity, cramp-like, with no irradiation and no aggravating or relieving factors. The pain was associated with bilious vomiting and no other associated digestive manifestations, notably no pruritus, jaundice, or abdominal distension, no externalized digestive hemorrhage, and no associated extra-digestive manifestations. All of this was evolving in a context of apyrexia and altered general condition, characterized by asthenia and weight loss, with no specific figures provided.

On examination, the patient was conscious (Glasgow Coma Scale (GCS) 15/15), normocardiac (heart rate (HR) 80 beats/minute), normotensive, and slightly tachypneic (respiratory rate 22 cycles/minute), apyretic (36°C), and had epigastric tenderness with no guarding. His blood results showed a hemoglobin level of 10.7 g/dL (normocytic normochromic anemia), a leukocyte count of 8130, a C-reactive protein level of 102, normal lipasemia, and his liver workup revealed biological cholestasis. His renal function was normal, with hyponatremia and normal potassium levels (Table 1), accompanied by high blood sugar levels.

**Table 1:** Laboratory findings in our case

Parameters	Patients Values	Reference Range
White blood cell count (/µL)	8130	4000–11,000
Eosinophilic count (/µL)	140	20–630
Lymphocytic count (/µL)	1770	1000–4800
Neutrophilic count (/µL)	5080	1400–7700
Hemoglobin (g/dL)	10.7	13–18
Lipase (IU/L)	44	13–78
Alanine aminotransferase (IU/L)	15	<65
Aspartate aminotransferase (IU/L)	25	<50
Alkaline phosphatase (IU/L)	169	40–129
Gamma-glutamyl transferase (IU/L)	128	8–61
Total bilirubinemia (µmol/L)	15	<17
Creatinine (µmol/L)	63	60–120
Natremia (mmol/L)	125	136–145
Kaliemia (mmol/L)	4.40	3.5–4.6
C-reactive protein (mg/L)	102	<5
Albumin (g/L)	27	35–50

**Figure 1:**
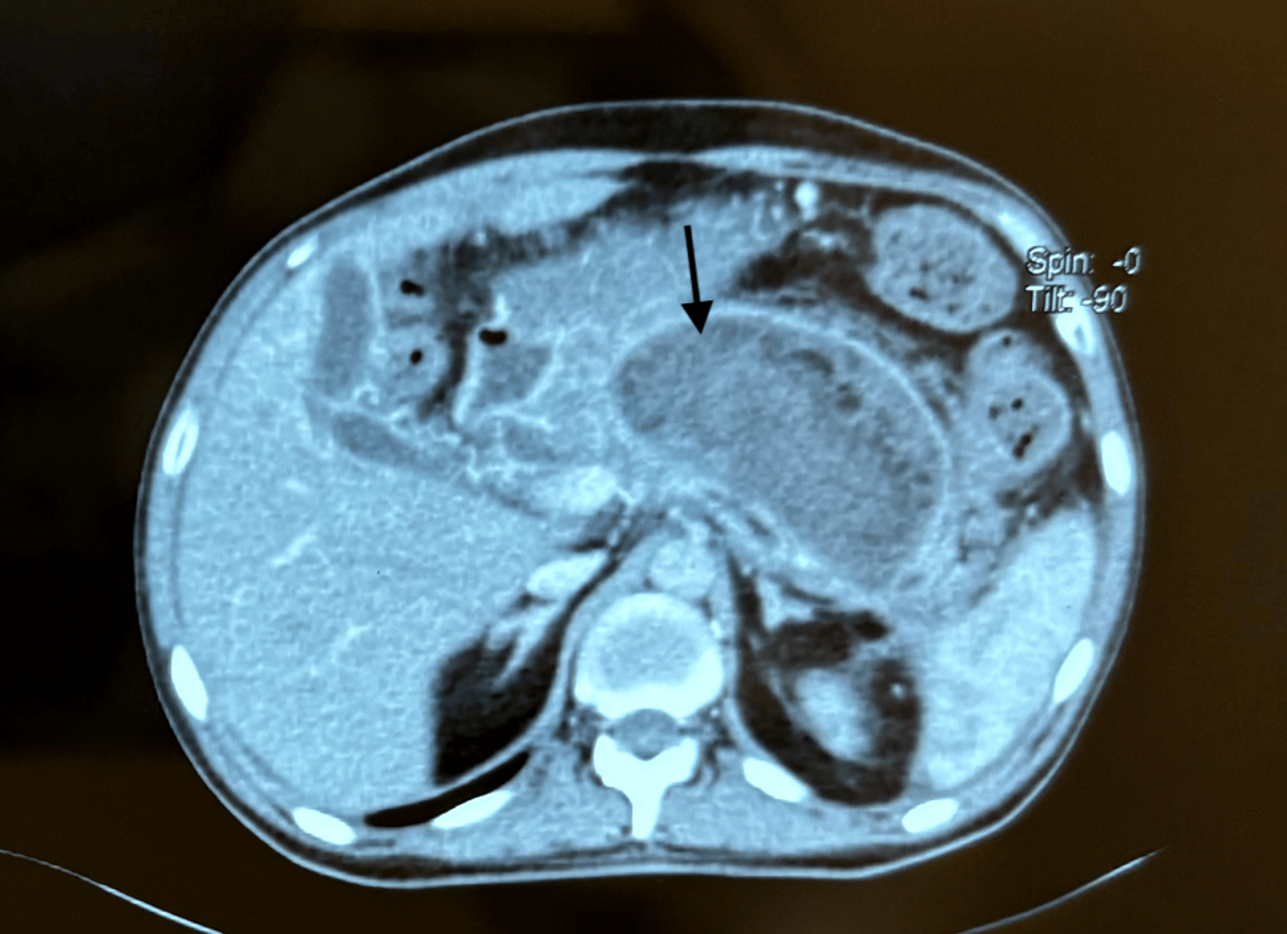
Abdominal CT scan in axial section with injection of contrast medium showing a pseudocyst of the pancreas (arrow).

He underwent an abdominal CT scan on hospital day one, which revealed a voluminous intrapancreatic cephalo-corporeo-caudal collection, occupying the posterior cavities of the epiplons and fusing at the level of the left parieto-colic angle, well limited, with heterogeneous hypoechoic content, with an enhanced wall after injection of contrast medium, measuring 12 x 7.5 x 8.3 cm (Figure 1).

**Figure 2:**
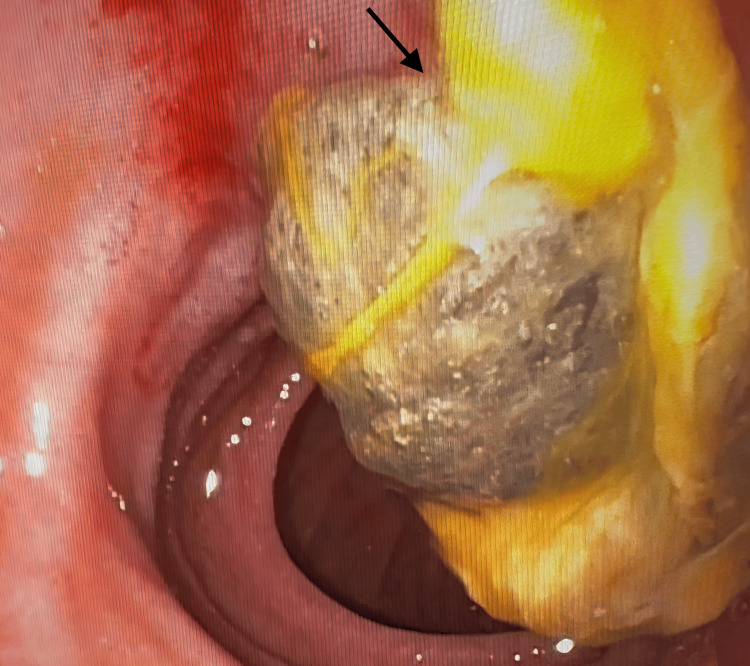
Photograph taken during the endoscopic procedure, showing fistulization of the pancreatic pseudocyst in the duodenum. The arrow indicates the fistulous opening with visible escape of necrotic debris from the pseudocyst into the duodenum.

The patient was scheduled for endoscopic drainage of the intrapancreatic collection on day four of hospitalization. During the procedure, an abnormal opening in the medial wall of the first portion of the duodenum (D1) with a mucus plug was fortuitously discovered (Figure 2).

A diagnosis of spontaneous pancreaticoduodenal fistula was made, and although drainage was established via the fistula, the cyst remained large, and the symptoms persisted.

**Figure 3:**
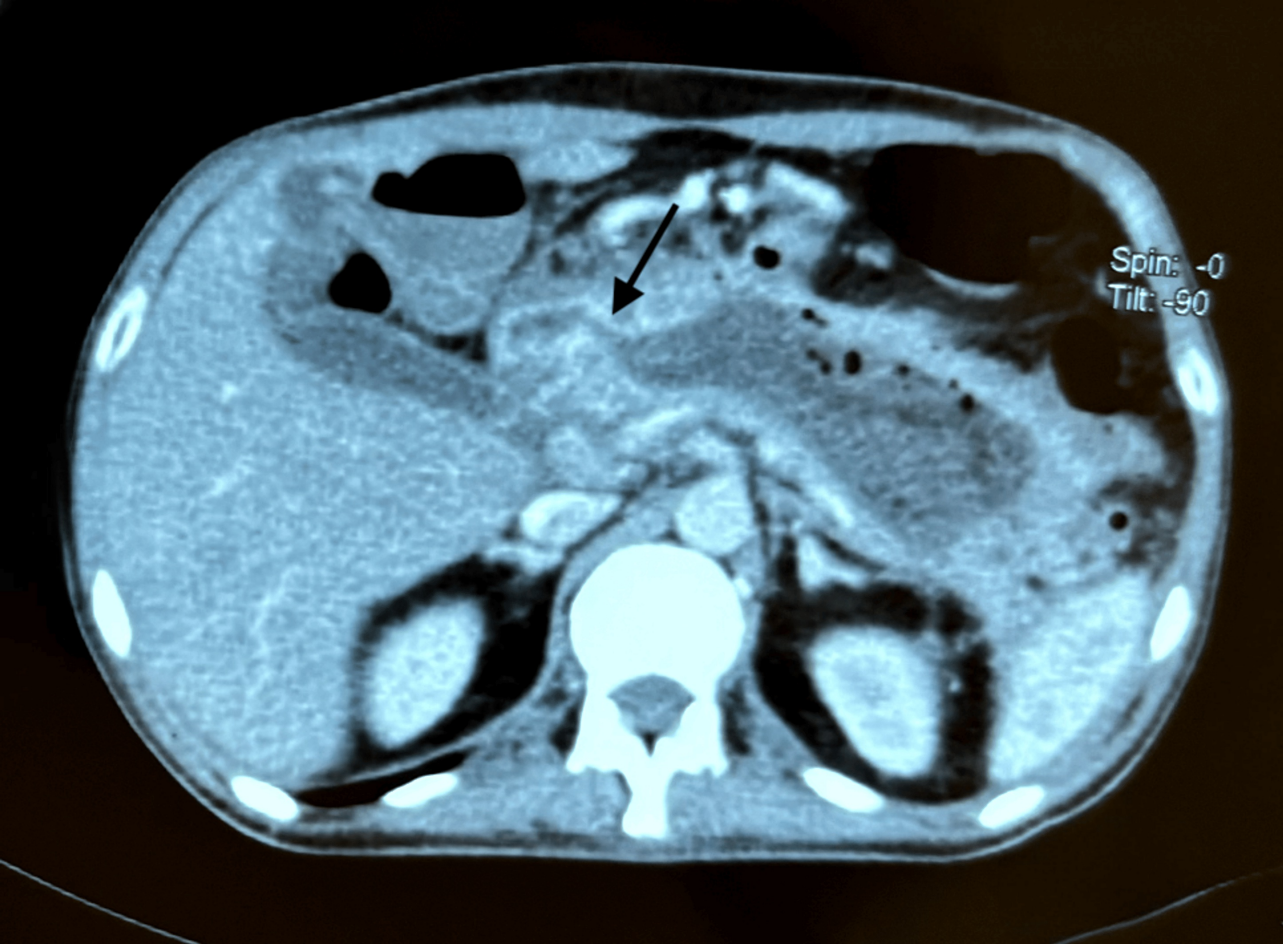
Abdominal CT scan in axial section showing an abnormal fistulous communication between a pancreatic pseudocyst and the first (D1) segment of the duodenum (arrow).

A follow-up abdominal CT scan was performed on day six of hospitalization. It showed a swollen pancreas with a voluminous collection occupying almost the entire pancreas, containing air bubbles, hypodense and heterogeneous, enhanced at the periphery after injection of contrast medium, measuring 15.2 x 43 x 59 mm. This collection communicates with the internal surface of the proximal part of the first portion of the duodenum (D1) through a fistulous path measuring 5.2 mm in maximum diameter (Figure 3), associated with regular circumferential parietal thickening of D1 and D2, enhanced on target after injection of contrast medium, measuring 8 mm thick with a reactive appearance. This collection appears to communicate with the pleura at the level of the left pleural cul-de-sac, accompanied by a small amount of left pleural effusion and passive atelectasis of the lung parenchyma on the opposite side.

On hospital day 10, the patient's condition worsened due to severe hyponatremia, which was refractory to correction. He developed neurological deterioration with altered consciousness and was transferred to the intensive care unit (ICU). Unfortunately, his condition continued to deteriorate, and he died shortly after ICU admission.

## Discussion

Spontaneous fistulization of pancreatic pseudocysts is a rare but recognized phenomenon, reported in less than 3% of cases [1]. It is most commonly described in adults [4] and may involve various sites, including the stomach, colon, duodenum, peritoneal cavity, pleural cavity, portal vein, or even the abdominal wall. The etiopathogenesis of this complication is generally attributed to the persistent necrotizing action of pancreatic enzymes on adjacent tissues [5]. Treatment of internal fistulization depends essentially on the location of the intra-abdominal organ concerned. Colon fistulization is a potentially life-threatening complication due to its association with intraperitoneal rupture, massive bleeding, and fulminant secretion [6].

A predominantly neutrophilic hyperleukocytosis in the blood count is often associated with a superinfected false pancreatic cyst. Hyperglycemia in false pancreatic cysts is variable and may indicate involvement of the endocrine pancreas [7].

Imaging (especially ultrasound and CT) is of fundamental importance for positive diagnosis, topography (including relationships and impact on neighboring organs), and monitoring. Ultrasound provides a true assessment of the lesion (location, number, structure, and relationships of the pseudocyst), guides the therapeutic indications, and monitors the evolution (resolution or complications) and maturation of the wall of the pseudocyst of the pancreas [8]. Its reliability varies from 75 to 93%. A CT scan can be used to diagnose a false pancreatic cyst with certainty and to determine its size, location, and relationship [9]. Endoscopic retrograde cholangio-pancreatography (ERCP) is not necessary for the diagnosis of false pancreatic cysts, but it can be used to demonstrate that they are communicating, to look for an obstruction in the Wirsung, to demonstrate pancreatic ductal lesions, and to look for a bile duct anomaly, whether or not associated with biliary lithiasis [8]. Although ERCP was not required in our case, it may be useful in complex presentations to evaluate ductal communication and guide the management of pancreatic pseudocysts [10].

Spontaneous regression occurs in approximately 40% of cases [11], with a higher frequency observed in acute pancreatitis compared to chronic calcifying pancreatitis. Several types of complications can arise. Infection of the pseudocyst, which occurs in approximately 10% of cases, is the most severe, with mortality rates ranging from 5% to 40% [12]. Intracystic hemorrhage secondary to erosion of the arterial walls is seen in 6-8% of false pancreatic cysts [13]. Rupture is a rare but serious complication, particularly when involving a pseudonecrotic cyst and in cases of intraperitoneal rupture. The mortality rate is approximately 80% in cases of hemoperitoneum and 15% in the absence of hemoperitoneum. Compression of neighboring organs is usually a chronic complication, especially in cases of large false pancreatic cysts, and primarily affects the duodenum and bile ducts [14].

From a therapeutic perspective, cysto-digestive shunts represent the treatment of choice, as they combine the lowest rates of immediate complications with the best long-term results. External surgical drainage often leads to the formation of an external pancreatic fistula and is associated with a mortality rate ranging from 22% to 55.5%, depending on the study [15]. The drainage duration typically spans 15 to 30 days, with recurrence rates ranging from 23% to 38%. Pancreatic resection is rarely performed due to its high associated mortality and morbidity [14].

Minimally invasive approaches such as endoscopic cystodigestive bypass are increasingly recognized as first-line treatments when anatomical contiguity exists between the pseudocyst and the digestive lumen, offering lower morbidity and mortality compared to surgery [16]. In our case, the direct fistulization into the duodenum allowed for endoscopic management, avoiding severe complications and eliminating the need for surgical intervention. Transpapillary endoscopic drainage is used for cysts communicating with the Wirsung duct or with a large collateral; this technique is inconsistently effective, but there is a significant risk of recurrence. Transcutaneous drainage of pancreatic pseudocysts has been proposed by some authors as a first-line treatment [17]. However, long-term outcomes reported in the literature remain highly variable.

A significant limitation of this report is its single-case design. While it provides valuable insights into the rare event of spontaneous pancreaticoduodenal fistulization, the findings are specific to this patient and may not be generalizable. The unique clinical presentation, disease course, and outcome may differ from those of other patients with similar conditions. Nonetheless, single-case reports remain crucial in guiding clinical suspicion and management in atypical or rare presentations.

## Conclusions

Although the diagnosis of pancreatic pseudocysts is generally straightforward with ultrasound and CT imaging, their management remains subject to debate. Traditionally, surgical drainage or bypass has been the standard approach, especially in complicated cases. However, advances in endoscopy have led to the development of less invasive alternatives such as cystodigestive bypass and transpapillary drainage. This case highlights that endoscopic management may be a safe and effective option in selected cases of spontaneous pseudocyst fistulization, potentially avoiding surgical morbidity.
